# Supplementary material for: Filamentous structures in the cell envelope are associated with bacteroidetes gliding machinery
Source: Commun Biol. 2023 Jan 23;6:94. doi: 10.1038/s42003-023-04472-3 (PMC9870892; doi:10.1038/s42003-023-04472-3)
Supplement: Supplementary file 2 — Supplementary Information [file 42003_2023_4472_MOESM2_ESM.pdf]

Supplementary information:

Filamentous Structures in the Cell Envelope Are Associated with Bacteroidetes Gliding Machinery

Satoshi Shibata, Yuhei O. Tahara, Eisaku Katayama, Akihiro Kawamoto, Takayuki Kato, Yongtao Zhu, Daisuke Nakane, Keiichi Namba, Makoto Miyata, Mark J. McBride, and Koji Nakayama

Supplementary Figures

Supplementary Figure 1. Identification and localization of proteins in the cell-surface protein fraction of wild type *F. johnsoniae* ATCC 17061.

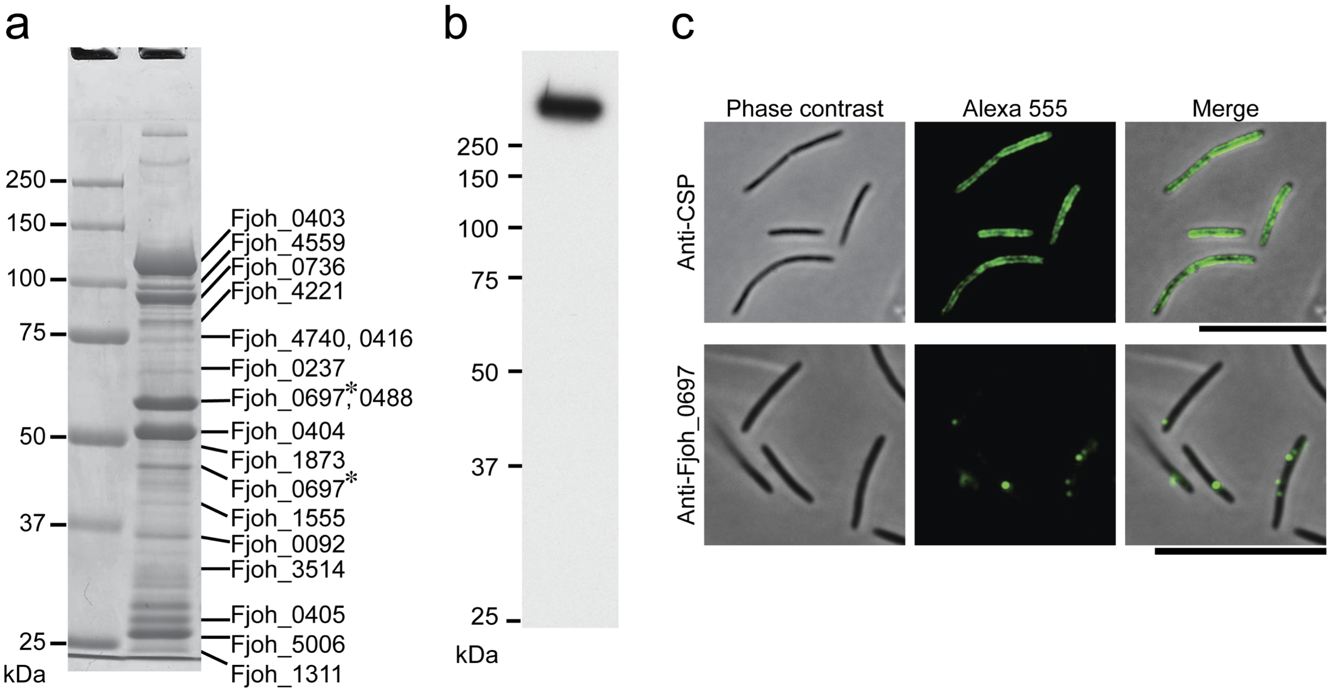

a Identification of cell-surface proteins. Cell-surface proteins were removed from the wild type cells by shearing force with passage through a 26 G-1/2 inch needle several times, collected by ultra-centrifugation after removal of cells, and subjected to SDS-PAGE. Each protein band on the gel was identified with peptide mass fingerprinting analysis using MALDI-TOF-MS. The asterisk indicates the Fjoh_0697 protein. **b** Immunoblot detection of SprB with anti-SprB antiserum in the cell-surface proteins. **c** Localization of the cell-surface proteins and Fjoh_0697 protein. Intact cells were reacted with antiserum raised against pooled cell-surface proteins (anti-CSP) or with anti-Fjoh_0697 antiserum, and Alexa Fluor 555-conjugated anti-rabbit IgG secondary antibodies were used to visualize the proteins. Bars indicate 10 µm. 3 technical replicates.

Supplementary Figure 2. Localization of SprB on non-elongated control and filamentous cells.

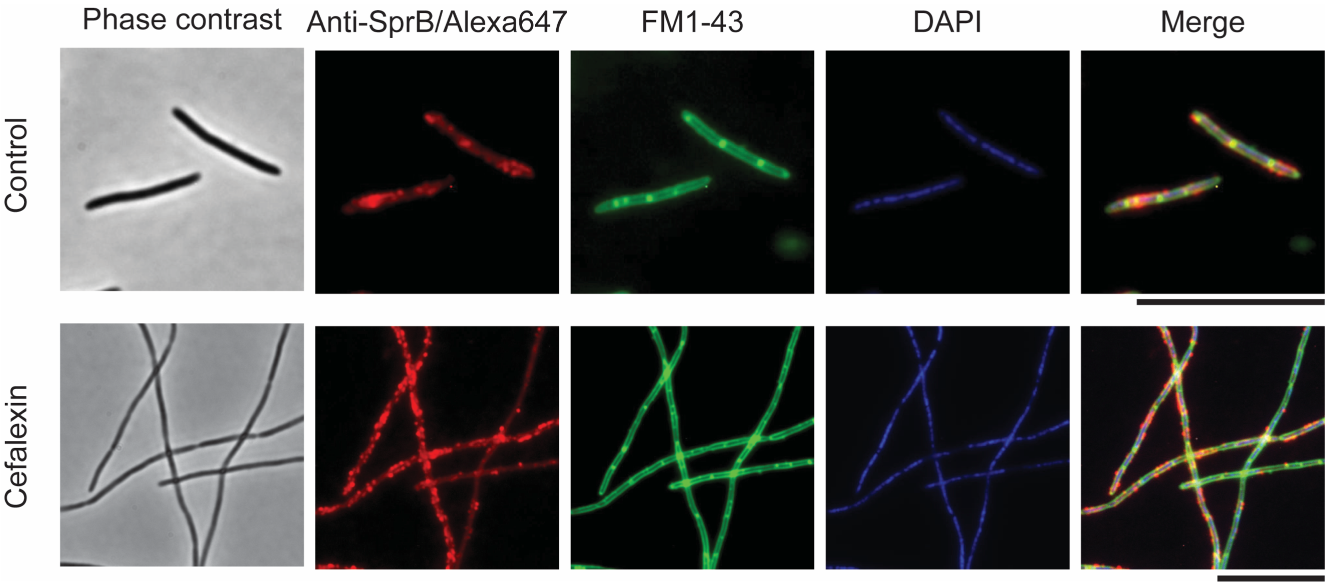

Cells were grown in the presence or absence of 20 μg per ml of cephalexin for 6 h and chemically fixed with 2% glutaraldehyde. SprB was labeled with 1:1000 dilution of antiserum against SprB for 30 min, followed by staining with Alexa Fluor 647-conjugated anti-rabbit IgG secondary antibody (red). Cell membrane and DNA were labeled with FM1-43 (green), and DAPI (blue), respectively. Bars indicate 10 μm.

Supplementary Figure 3. Presence or absence of multi-rail structures in *gld*, *spr* and *remA* mutants.

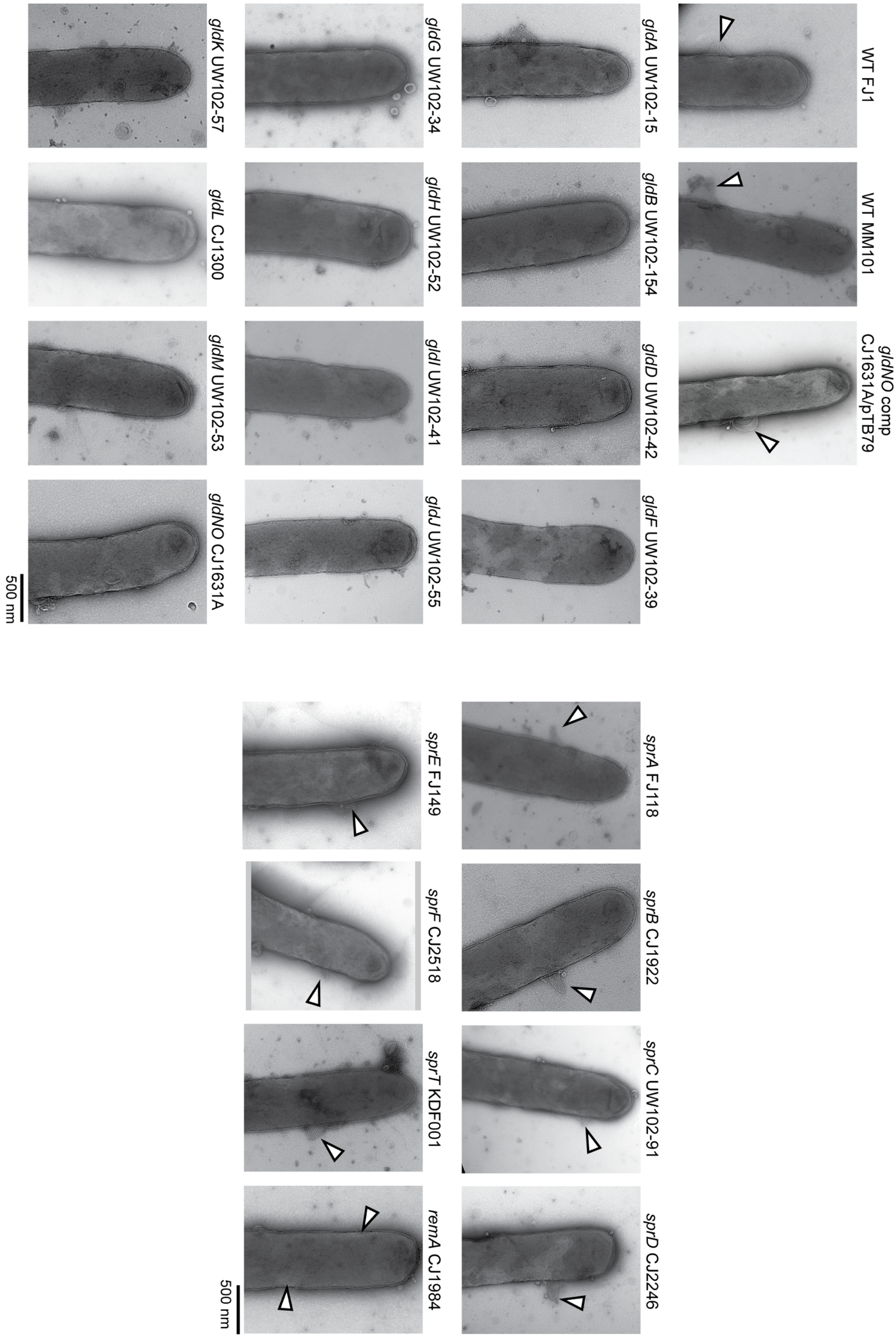

Osmotically shocked cells of various *gld* and *spr* mutants were stained with 0.5% uranyl acetate and observed by TEM. Arrowheads denote the multi-rail structures. Bars indicate 500 nm.

Supplementary Figure 4. Uncropped images of the immuno blots and gel.

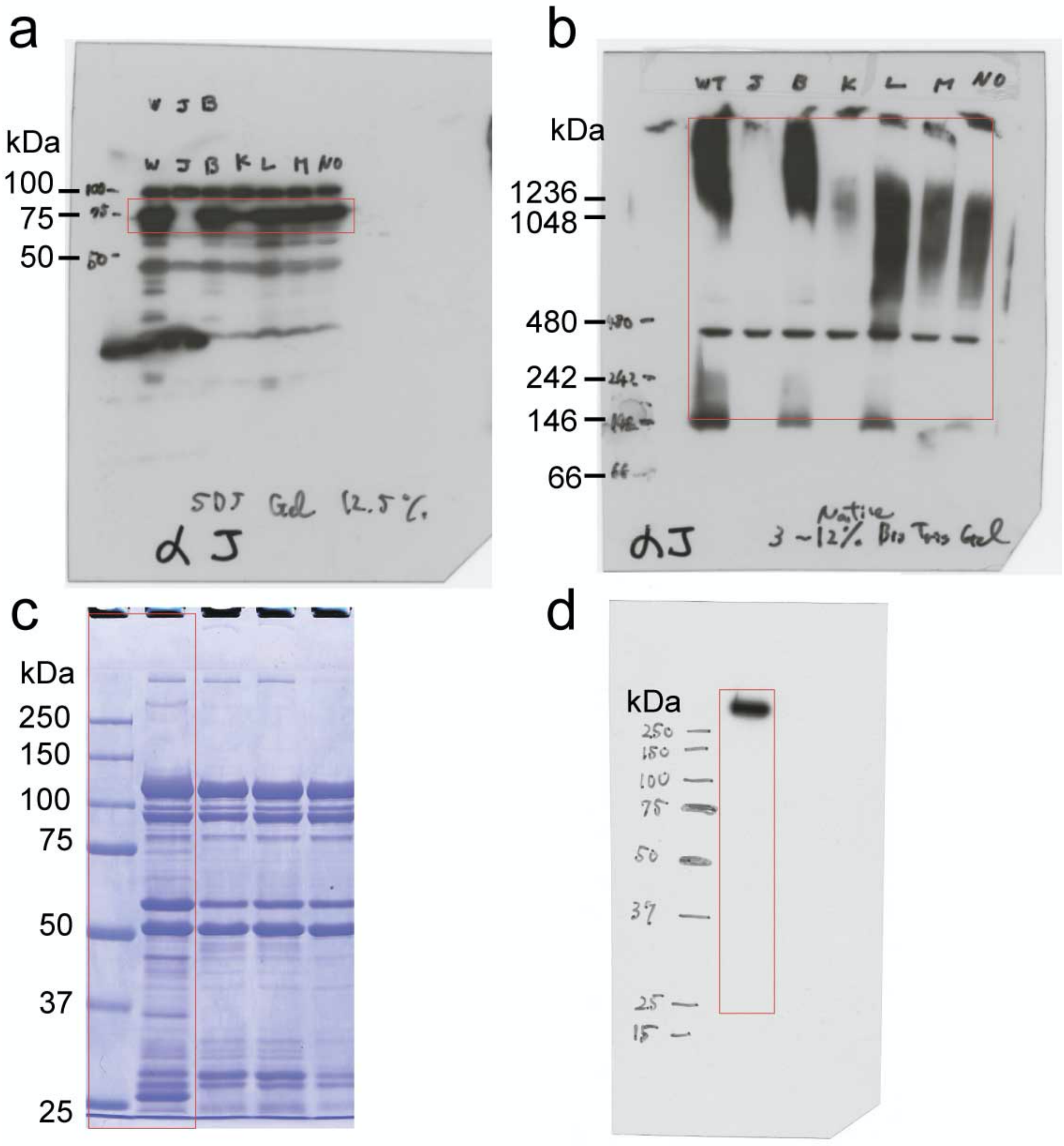

a and **b** Unprocessed images of immunoblot with anti GldJ antiserum for Figure 5b and 5c respectively. **c** Unprocessed Coomassie brilliant blue (CBB)-stained SDS-PAGE gel image for Supplementary Figure 1a. **d** Unprocessed image of immunoblot with anti-SprB antiserum for Supplementary Figure 1b. Image areas used in the figures are indicated by red boxes.

Supplementary Table 1. Identification of proteins in the cell surface fraction

Locus tag	Predicted function and domain	Amino acids	Observed MM (kDa)	Presence (Y) or absence (N) of signal sequence	Predicted location*
Fjoh_0092	Hypothetical lipoprotein	355	37	Y	E
Fjoh_0237	Hypothetical protein	635	68	Y	OM, P, E
Fjoh_0403	SusC-likeTonB-dependent receptor	1083	110	Y	OM
Fjoh_0404	RagB/SusD domain protein	488	51	Y	E, OM
Fjoh_0405	Hypothetical lipoprotein	272	28	Y	E
Fjoh_0416	Peptidase family S46; dipeptidyl-peptidase 7	714	76	Y	P
Fjoh_0488	TPR repeat-containing protein	556	59	Y	P
Fjoh_0697	OmpA/MotB domain protein	487	59, 42	Y	P, OM
Fjoh_0736	SusC-like TonB-dependent receptor	942	90	Y	OM
Fjoh_1311	Hypothetical protein	308	24	Y	OM
Fjoh_1555	T9SS protein PorV	402	40	Y	OM
Fjoh_1873	PKD domain containing protein	519	51	N	E
Fjoh_3514	Hypothetical protein	301	35	Y	E
Fjoh_4221	TonB-dependent siderophore recepto	795	80	Y	OM
Fjoh_4559	SusC-like TonB-dependent receptor CusC1 involved in chitin utilization	1010	100	Y	OM
Fjoh_4740	Hypothetical protein	972	76	N	C
Fjoh_5006	Hypothetical lipoprotein	266	25	Y	E

* E, extracellular; OM, outer membrane; P, periplasm; C, cytoplasm.

Supplementary Table 2. Bacterial strain list including presence or absence of the multi-rail structure

Bacterial strain	Mutation	Presence (Y) or absence (N) of the multi-rail structure	References
F. johnsoniae			
ATCC17061 (UW101)	Wild type	Y	(3, 4)
FJ1	Wild type	Y	(3)
MM101	Wild type	Y	(3)
CJ1827	Wild type (*rpsL2*)	Y	(1)
UW102-15	*gldA*	N	(2)
UW102-154	*gldB*	N	(5)
UW102-42	*gldD*	N	(6)
UW102-39	*gldF*	N	(7)
UW102-34	*gldG*	N	(7)
UW102-52	*gldH*	N	(8)
UW102-41	*gldI*	N	(3)
UW102-55	*gldJ*	N	(9)
UW102-57	*gldK*	N	(10, 11)
CJ1300	*gldL*	N	(11)
UW102-53	*gldM*	N	(11)
CJ1631A	*gldNO*	N	(12)
FJ118	*sprA*	Y	(13)
CJ1922	*sprB*	Y	(1)
UW102-91	*sprC*	Y	(14)
CJ2246	*sprD*	Y	(This study)
FJ149	*sprE*	Y	(15)
CJ2518	*sprF*	Y	(This study)
KDF001	*sprT*	Y	(16)
CJ1984	*remA*	Y	(17)
CJ1640	Complemented strain of *gldNO* (CJ1631A with pTB79 carrying *gldN*)	Y	(12)
S. grandis str. Lewin	Wild type	Y	(18)

Supplementary Table 3. *gld*, *spr* and *remA* orthologs in *S. grandis*

	F.johnsoniae	*S. grandis*	E-val*
gldA	Fjoh_1516	SGRA_2200	1.00E-99
gldB	Fjoh_1793	SGRA_1680	2.00E-25
gldD	Fjoh_1540	SGRA_2175	5.00E-22
gldF	Fjoh_2722	SGRA_0089	1.00E-72
gldG	Fjoh_2721	SGRA_4171	4.00E-86
gldH	Fjoh_0890	SGRA_2230	2.00E-77
gldI	Fjoh_2369	-[†]	-
gldJ	Fjoh_1557	SGRA_3016	4.00E-119
gldK	Fjoh_1853	SGRA_1590	3.00E-110
gldL	Fjoh_1854	SGRA_1587	7.00E-15
gldM	Fjoh_1855	SGRA_1586	5.00E-25
gldN	Fjoh_1856	SGRA_1585<br>SGRA_1584<br>SGRA_2587	8.00E-27<br>8.00E-26<br>1.00E-15
sprA	Fjoh_1653	SGRA_2672	0.0
sprB	Fjoh_0979	SGRA_2693<br>SGRA_0732<br>SGRA_3496<br>SGRA_3915	5.00E-111<br>1.00E-95<br>3.00E-81<br>8.00E-45
sprC	Fjoh_0981	SGRA_0445	6.00E-09
sprD	Fjoh_0980	SGRA_0444	5.00E-06
sprE	Fjoh_1051	SGRA_1045	2.00E-35
sprF	Fjoh_0978	SGRA_0444<br>SGRA_2554<br>SGRA_1711<br>SGRA_0061<br>SGRA_3015<br>SGRA_1591	4.00E-10<br>1.00E-08<br>7.00E-06<br>7.00E-05<br>7.00E-05<br>1.00-04
sprT	Fjoh_1466	SGRA_0916	5.00E-20
remA	Fjoh_0808	-	-

*E-values were calculated by BLASTP.

[†] (-) indicates that ortholog was not identified in *S. grandis* genome.

Supplementary References

1. Rhodes, R.G., Pucker, H.G. & McBride, M.J. Development and use of a gene deletion strategy for *Flavobacterium johnsoniae* to identify the redundant gliding motility genes *remF*, *remG*, *remH*, and *remI*. *J. Bacteriol.* **193**, 2418-2428 (2011).
2. Agarwal, S., Hunnicutt, D.W. & McBride, M.J. Cloning and characterization of the *Flavobacterium johnsoniae* (*Cytophaga johnsonae*) gliding motility gene, gldA. *Proc. Natl. Acad. Sci. USA* **94**, 12139-12144 (1997).
3. McBride, M.J. & Braun, T.F. GldI is a lipoprotein that is required for *Flavobacterium johnsoniae* gliding motility and chitin utilization. *J. Bacteriol.* **186**, 2295-2302 (2004).
4. McBride, M.J. *et al.* Novel features of the polysaccharide-digesting gliding bacterium *Flavobacterium johnsoniae* as revealed by genome sequence analysis. *Appl. Environ. Microbiol.* **75**, 6864-6875 (2009).
5. Hunnicutt, D.W. & McBride, M.J. Cloning and characterization of the *Flavobacterium johnsoniae* gliding-motility genes *gldB* and *gldC*. *J. Bacteriol.* **182**, 911-918 (2000).
6. Hunnicutt, D.W. & McBride, M.J. Cloning and characterization of the *Flavobacterium johnsoniae* gliding motility genes *gldD* and *gldE*. *J. Bacteriol.* **183**, 4167-4175 (2001).
7. Hunnicutt, D.W., Kempf, M.J. & McBride, M.J. Mutations in *Flavobacterium johnsoniae gldF* and *gldG* disrupt gliding motility and interfere with membrane localization of GldA. *J. Bacteriol.* **184**, 2370-2378 (2002).
8. McBride, M.J., Braun, T.F. & Brust, J.L. *Flavobacterium johnsoniae* GldH is a lipoprotein that is required for gliding motility and chitin utilization. *J. Bacteriol.* **185**, 6648-6657 (2003).
9. Braun, T.F. & McBride, M.J. *Flavobacterium johnsoniae* GldJ is a lipoprotein that is required for gliding motility. *J. Bacteriol.* **187**, 2628-2637 (2005).
10. Chang, L.E., Pate, J.L. & Betzig, R.J. Isolation and characterization of nonspreading mutants of the gliding bacterium *Cytophaga johnsonae*. *J. Bacteriol.* **159**, 26-35 (1984).

11. Braun, T.F., Khubbar, M.K., Saffarini, D.A. & McBride, M.J. *Flavobacterium johnsoniae* gliding motility genes identified by mariner mutagenesis. *J. Bacteriol.* **187**, 6943-6952 (2005).
12. Rhodes, R.G. *et al. Flavobacterium johnsoniae gldN* and *gldO* are partially redundant genes required for gliding motility and surface localization of SprB. *J. Bacteriol.* **192**, 1201-1211 (2010).
13. Nelson, S.S., Glocka, P.P., Agarwal, S., Grimm, D.P. & McBride, M.J. *Flavobacterium johnsoniae* SprA is a cell surface protein involved in gliding motility. *J. Bacteriol.* **189**, 7145-7150 (2007).
14. Rhodes, R.G., Nelson, S.S., Pochiraju, S. & McBride, M.J. *Flavobacterium johnsoniae sprB* is part of an operon spanning the additional gliding motility genes *sprC*, *sprD*, and *sprF*. *J. Bacteriol.* **193**, 599-610 (2011).
15. Rhodes, R.G., Samarasam, M.N., Van Groll, E.J. & McBride, M.J. Mutations in *Flavobacterium johnsoniae sprE* result in defects in gliding motility and protein secretion. *J. Bacteriol.* **193**, 5322-5327 (2011).
16. Sato, K. *et al.* A protein secretion system linked to bacteroidete gliding motility and pathogenesis. *Proc. Natl. Acad. Sci. USA* **107**, 276-281 (2010).
17. Shrivastava, A., Rhodes, R.G., Pochiraju, S., Nakane, D. & McBride, M.J. *Flavobacterium johnsoniae* RemA is a mobile cell surface lectin involved in gliding. *J. Bacteriol.* **194**, 3678-3688 (2012).
18. Saw, J.H. *et al.* Complete genome sequencing and analysis of *Saprospira grandis* str. Lewin, a predatory marine bacterium. *Stand. Genomic Sci.* **6**, 84-93 (2012).
