# Supplementary material for: Filamentous structures in the cell envelope are associated with bacteroidetes gliding machinery
Source: Commun Biol. 2023 Jan 23;6:94. doi: 10.1038/s42003-023-04472-3 (PMC9870892; doi:10.1038/s42003-023-04472-3)
Supplement: Supplementary file 3 — Description of Additional Supplementary Files [file 42003_2023_4472_MOESM3_ESM.pdf]

Description of Additional Supplementary Files

File name: Supplementary Movie 1

Description: Gliding cells labeled with antiCSP antiserum and Alexa Fluor 555-conjugated secondary antibody and imaged by TIRF microscopy.

File name: Supplementary Movie 2

Description: A gliding cell with a type I signal located at a position a fixed distance away from a cell pole. The cell was labeled with anti-CSP antiserum and Alexa Fluor 555- conjugated secondary antibody and imaged by TIRF microscopy.

File name: Supplementary Movie 3

Description: A gliding cell with a type II signal showing left-handed helical movement on the gliding cell. Cell was labeled with anti-CSP antiserum and Alexa Fluor 555- conjugated secondary antibody and imaged by TIRF microscopy.

File name: Supplementary Movie 4

Description: A gliding cell labeled with antiFjoh_0697 antiserum and Alexa Fluor 555- conjugated secondary antibody and imaged by TIRF microscopy. Related to Figure 1a.

File name: Supplementary Movie 5

Description: Left-handed helical movement of SprB on a gliding cell imaged by TIRF microscopy. SprB was labeled with anti-SprB antiserum and Alexa Fluor 555-conjugated secondary antibody. Related to Figure 1b.

File name: Supplementary Movie 6

Description: U-turn movement of SprB on a gliding cell imaged by immunofluorescence microscopy. SprB was labeled with anti-SprB antiserum and Alexa Fluor 555-conjugated secondary antibody. Related to Figure 2a.

File name: Supplementary Movie 7

Description: Passing movement of SprB on a gliding cell imaged by immunofluorescence microscopy. Related to Figure 2b.

File name: Supplementary Movie 8

Description: Passing movement of SprB on a cephalexin-treated filamentous cell imaged by immunofluorescence microscopy. Related to Figure 2c.

File name: Supplementary Movie 9

Description: Slowdown-and-stop movement of SprB on a cephalexin-treated filamentous cell imaged by immunofluorescence microscopy. Related to Figure 2d and 2f.

File name: Supplementary Movie 10

Description: Stay-and-go movement of SprB on a cephalexin-treated filamentous cell imaged by immunofluorescence microscopy. Related to Figure 2e.

File name: Supplementary Movie 11

Description: Cryo-electron tomography with a wild-type cell (a). Bar indicates 200 nm. Related to Figure 4a and 4c.

File name: Supplementary Movie 12

Description: Cryo-electron tomography with a wild-type cell (b). Bar indicates 200 nm. Related to Figure 4b and 4d.

File name: Supplementary Movie 13

Description: Cryo-electron tomography with a gldJ mutant cell. Bar indicates 200 nm.

File name: Supplementary Movie 14

Description: Cryo-electron tomography with a gldK mutant cell. Bar indicates 200 nm.

File name: Supplementary Movie 15

Description: Cryo-electron tomography with a gldL mutant cell. Bar indicates 200 nm.

File name: Supplementary Movie 16

Description: Cryo-electron tomography with a gldM mutant cell. Bar indicates 200 nm.

File name: Supplementary Movie 17

Description: Cryo-electron tomography with a gldNO mutant cell. Bar indicates 200 nm.

File name: Supplementary Movie 18

Description: Cryo-electron tomography with a S. grandis cell. Bar indicates 200 nm.

File name: Supplementary Data 1

Description: The source data behind the graphs in Figure 1d, 1e, and 1f.

File name: Supplementary Data 2

Description: The source data behind the graphs in Figure 2g.
