# Supplementary material for: Filamentous structures in the cell envelope are associated with bacteroidetes gliding machinery
Source: Commun Biol. 2023 Jan 23;6:94. doi: 10.1038/s42003-023-04472-3 (PMC9870892; doi:10.1038/s42003-023-04472-3)
Supplement: Supplementary file 24 — Reporting Summary [file 42003_2023_4472_MOESM24_ESM.pdf]

COMMSBIO-22-3575B

Corresponding author(s): Satoshi Shibata and Koji Nakayama

Last updated by author(s): Dec 27, 2022

Reporting Summary

Nature Portfolio wishes to improve the reproducibility of the work that we publish. This form provides structure for consistency and transparency in reporting. For further information on Nature Portfolio policies, see our Editorial Policies and the Editorial Policy Checklist.

Statistics

For all statistical analyses, confirm that the following items are present in the figure legend, table legend, main text, or Methods section.

n/a	Confirmed	
☐	☒	The exact sample size (*n*) for each experimental group/condition, given as a discrete number and unit of measurement
☐	☒	A statement on whether measurements were taken from distinct samples or whether the same sample was measured repeatedly
☒	☐	The statistical test(s) used AND whether they are one- or two-sided<br>*Only common tests should be described solely by name; describe more complex techniques in the Methods section.*
☒	☐	A description of all covariates tested
☒	☐	A description of any assumptions or corrections, such as tests of normality and adjustment for multiple comparisons
☐	☒	A full description of the statistical parameters including central tendency (e.g. means) or other basic estimates (e.g. regression coefficient) AND variation (e.g. standard deviation) or associated estimates of uncertainty (e.g. confidence intervals)
☒	☐	For null hypothesis testing, the test statistic (e.g. *F*, *t*, *r*) with confidence intervals, effect sizes, degrees of freedom and *P* value noted<br>*Give P values as exact values whenever suitable.*
☒	☐	For Bayesian analysis, information on the choice of priors and Markov chain Monte Carlo settings
☒	☐	For hierarchical and complex designs, identification of the appropriate level for tests and full reporting of outcomes
☒	☐	Estimates of effect sizes (e.g. Cohen's *d*, Pearson's *r*), indicating how they were calculated

Our web collection on statistics for biologists contains articles on many of the points above.

Software and code

Policy information about availability of computer code

Data collection	MetaVue software (Molecular Device, CA, USA) was used for optical microscopy data acquisition. Xplore 3D software package (FEI) was used for cryo-EM data acquisition.
Data analysis	Microscopy data were analyzed with ImageJ 1.48r. 3D reconstructions were calculated using the IMOD software package. Surface-rendering images of cryo-Tomography were obtained using the three-dimensional modeling software Amira 5.2.2 (Visage Imaging, San Diego, CA). The schematic models of gliding machinery (Fig. 6) were created with Blender 2.81.

For manuscripts utilizing custom algorithms or software that are central to the research but not yet described in published literature, software must be made available to editors and reviewers. We strongly encourage code deposition in a community repository (e.g. GitHub). See the Nature Portfolio guidelines for submitting code & software for further information.

Data

Policy information about availability of data

All manuscripts must include a data availability statement. This statement should provide the following information, where applicable:

- Accession codes, unique identifiers, or web links for publicly available datasets
- A description of any restrictions on data availability
- For clinical datasets or third party data, please ensure that the statement adheres to our policy

The data sets generated and/or analyzed in this study are available from the corresponding authors upon reasonable request.

Human research participants

Policy information about studies involving human research participants and Sex and Gender in Research.

Reporting on sex and gender	This does not apply to our expriments.
Population characteristics	This does not apply to our expriments.
Recruitment	This does not apply to our expriments.
Ethics oversight	This does not apply to our expriments.

Note that full information on the approval of the study protocol must also be provided in the manuscript.

Field-specific reporting

Please select the one below that is the best fit for your research. If you are not sure, read the appropriate sections before making your selection.

☒ Life sciences ☐ Behavioural & social sciences ☐ Ecological, evolutionary & environmental sciences

For a reference copy of the document with all sections, see nature.com/documents/nr-reporting-summary-flat.pdf

Life sciences study design

All studies must disclose on these points even when the disclosure is negative.

Sample size	Sample sizes were chosen according to previous experience in similar experimental setups. Data collection was conducted multiple times (at least 3 technical replicates). Sample size and standard deviation were indicated in main text and figure section.
Data exclusions	None
Replication	Data collection was conducted multiple times (at least 3 technical replicates).
Randomization	Images were collected randomly in each set of experiments.
Blinding	The researchers were not blinded to sample identity.

Reporting for specific materials, systems and methods

We require information from authors about some types of materials, experimental systems and methods used in many studies. Here, indicate whether each material, system or method listed is relevant to your study. If you are not sure if a list item applies to your research, read the appropriate section before selecting a response.

Materials & experimental systems

n/a	Involved in the study	
☐	☒	Antibodies
☒	☐	Eukaryotic cell lines
☒	☐	Palaeontology and archaeology
☐	☒	Animals and other organisms
☒	☐	Clinical data
☒	☐	Dual use research of concern

Methods

n/a	Involved in the study	
☒	☐	ChIP-seq
☒	☐	Flow cytometry
☒	☐	MRI-based neuroimaging

Antibodies

Antibodies used	Rabbit anti SprB antiserum and rabbit anti GldJ antibody were provided by Dr. McBrided. Rabbit anti-Fjoh_0697 and anti-CSP antisera were generated in this study. Alexa Fluor 555-conjugated antibody against rabbit IgG (Abcam, ab150078). Goat anti-rabbit IgG conjugated to 5 nm diameter gold particles (BBI solutions,EM.GAR5/1).
Validation	The primary antibodies were validated by comparing a mutants sample that did not contain target protein to WT cell sample. Binding of Anti-Fjoh_0697 and anti-CSP antisera to cell surface were validated by fluorescence microscopy (Supplementary Figure 1).

Animals and other research organisms

Policy information about studies involving animals; ARRIVE guidelines recommended for reporting animal research, and Sex and Gender in Research

Laboratory animals	Bacterial strains used in this study were listed in the Supplementary table 2.
Wild animals	This does not apply to our expriments.
Reporting on sex	This does not apply to our expriments.
Field-collected samples	This does not apply to our expriments.
Ethics oversight	This does not apply to our expriments.

Note that full information on the approval of the study protocol must also be provided in the manuscript.
